# Brain-Derived Neurotrophic Factor in Pediatric Acquired Brain Injury and Recovery

**DOI:** 10.3390/biom14020191

**Published:** 2024-02-04

**Authors:** Amery Treble-Barna, Bailey A. Petersen, Zachary Stec, Yvette P. Conley, Ericka L. Fink, Patrick M. Kochanek

**Affiliations:** 1 Department of Physical Medicine & Rehabilitation, University of Pittsburgh School of Medicine, Pittsburgh, PA 15213, USA; bap87@pitt.edu (B.A.P.); zts20@pitt.edu (Z.S.); 2 Safar Center for Resuscitation Research, University of Pittsburgh School of Medicine, Pittsburgh, PA 15213, USA; finkel@ccm.upmc.edu (E.L.F.); kochanekpm@pitt.edu (P.M.K.); 3 Department of Health Promotion & Development, University of Pittsburgh School of Nursing, Pittsburgh, PA 15213, USA; yconley@pitt.edu; 4 Department of Critical Care Medicine, University of Pittsburgh School of Medicine, Pittsburgh, PA 15213, USA

**Keywords:** pediatric acquired brain injury, biomarkers, recovery, brain-derived neurotrophic factor

## Abstract

We review emerging preclinical and clinical evidence regarding brain-derived neurotrophic factor (BDNF) protein, genotype, and DNA methylation (DNAm) as biomarkers of outcomes in three important etiologies of pediatric acquired brain injury (ABI), traumatic brain injury, global cerebral ischemia, and stroke. We also summarize evidence suggesting that BDNF is (1) involved in the biological embedding of the psychosocial environment, (2) responsive to rehabilitative therapies, and (3) potentially modifiable. BDNF’s unique potential as a biomarker of neuroplasticity and neural repair that is reflective of and responsive to both pre- and post-injury environmental influences separates it from traditional protein biomarkers of structural brain injury with exciting potential to advance pediatric ABI management by increasing the accuracy of prognostic tools and informing clinical decision making through the monitoring of therapeutic effects.

## 1. Introduction

Pediatric acquired brain injury (ABI) is a leading cause of death and disability in children [1,2,3,4]. Traumatic brain injury (TBI) comprises the majority of pediatric ABI, with an incidence of 14.8 per 100,000 children per year in the United States. ABI also includes non-traumatic brain injury due to infection (4.3 per 100,000), stroke (2.4 per 100,000), and global cerebral ischemia due to cardiac arrest (1.3 per 100,000) [3]. In an international point prevalence study of children aged 7 days to 17 years old with ABI admitted to a pediatric intensive care unit, the most common were due to cardiac arrest (23%) and TBI (19%) [4]. Notably, children with cardiac arrest and children with TBI had the highest mortality (24%) and morbidity (49% unfavorable outcomes), respectively. 

The breadth and quality of evidence available to guide clinical management after pediatric ABI are disproportionately low relative to its medical and societal burden, which is driven by long-term neurobehavioral impairments [5,6,7,8,9]. Recovery from pediatric ABI is determined by the interaction of a multitude of dynamic biological, psychosocial, and therapeutic factors. This complexity results in a marked heterogeneity in outcomes and is cited as the most critical barrier to the development of accurate prognostic models and effective therapies [10,11,12].

The field has turned to biomarkers as one potential tool to explain this heterogeneity; however, the focus to date on protein biomarkers of structural brain injury (e.g., glial fibrillary acidic protein [GFAP], S100 calcium binding protein B [S100B], ubiquitin C-terminal hydrolase-L1 [UCH-L1]) will likely be inadequate to fully capture the complexity of factors that influence recovery [13,14,15]. The discovery of dynamic and potentially modifiable biomarkers that (a) reflect the child’s psychosocial environment (e.g., social determinants of health) and (b) respond to injury progression and recovery processes over days to months post injury would provide critical information about the biologic complexity underlying recovery. In the future, this information could revolutionize pediatric ABI management by identifying novel targets for therapy development, improving prognostic tools, and aiding therapeutic clinical decision making.

Brain-derived neurotrophic factor (BDNF) may be such a biomarker. BDNF is a well-studied member of the neurotrophin family of growth factors. Released pre- and post-synaptically from neurons, it mediates apoptosis, neuronal differentiation, cell survival, and synaptic strengthening [16,17]. Thus, in contrast to traditional ABI biomarkers of structural brain injury, BDNF is a biomarker of neuroplasticity and repair, essential to brain development, neuronal survival, and complex cognitive functions [18,19,20,21,22,23,24,25].

While biomarker studies often measure BDNF protein concentrations in the brain or periphery, upstream genetic and epigenetic influences on BDNF expression are also potentially informative. A single nucleotide polymorphism (SNP) producing a valine-to-methionine substitution at codon 66 (Val66Met; rs6265) in the *BDNF* gene is associated with the reduced activity-dependent secretion of BDNF [26]. Val66Met allele status, and especially possession of the Met allele, is associated with variations in brain structure and function, including smaller brain volumes [27,28,29,30] and lower connectivity [31,32], poorer neuropsychological functioning [33,34], and an increased risk for psychiatric and neurological conditions, in non-brain-injured individuals [35,36].

Epigenetics, in contrast, involves potentially heritable biochemical processes that regulate gene expression without altering the corresponding primary DNA sequence [37]. What is unique about epigenetic biomarkers that has great potential ramifications for ABI is that, through epigenetic processes, the biological and social environments of an individual impact when and to what extent genes are expressed within each cell type. The most investigated epigenetic modification is DNA methylation (DNAm), which involves the addition of a methyl group to cytosine–guanine dinucleotides (CpG). Higher DNAm in CpG rich promoters or gene regulatory regions is usually (but not always) associated with lower gene expression [38]. While epigenetic modifications in *BDNF* have been frequently investigated in association with brain-related phenotypes [39], the study of their potential as biomarkers of ABI recovery is just beginning to emerge.

Thus, herein, we review evidence from both preclinical and clinical studies of BDNF in pediatric ABI, focusing on three key insults, namely, TBI, global cerebral ischemia, and stroke, suggesting that peripheral BDNF concentrations, genotype, and DNAm may be markers of survival and recovery. We also review emerging evidence suggesting that BDNF is (1) involved in the biological embedding of the psychosocial environment, (2) responsive to rehabilitative therapies, and (3) potentially modifiable. These features support BDNF’s unique potential as a biomarker of neuroplasticity and neural repair that is reflective of and responsive to both pre- and post-injury environmental influences with exciting potential to advance pediatric ABI management by increasing the accuracy of prognostic tools and informing therapeutic decision making through its use as an intervention response biomarker of therapeutic effects (i.e., a pharmacodynamic response biomarker).

## 2. BDNF in Preclinical Models of Pediatric ABI

**Figure 1:**
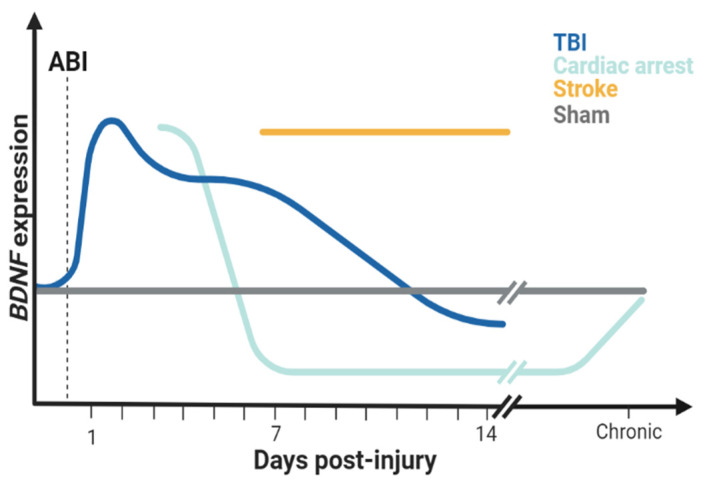
Brain-derived neurotrophic factor (BDNF) expression after acquired brain injury (ABI) in preclinical models in developing animals. Animals with traumatic brain injury (TBI, blue) have higher BDNF expression compared with animals with the sham operation (gray) on the first day after injury, with some studies showing higher BDNF expression up to 7 days after TBI vs. sham followed by lower BDNF expression at 14 days post injury vs. sham. In pediatric models of global ischemia produced by cardiac arrest (aqua), BDNF is higher than that of sham animals at 4 days after injury, followed by lower BDNF expression vs. sham at 7 days after injury. BDNF then increases to near-sham concentrations at 30 days post injury. Pediatric stroke models (orange) have higher BDNF expression vs. sham animals at 7 and 14 days after injury. Missing sections indicate a lack of available preclinical studies in models of ABI in developing animals during these time periods.

Preclinical studies of brain injury provide unique insight into the role of BDNF in neuroplasticity and repair (see Table 1). In animal models, the BDNF response to brain injury is dynamic in the days and weeks after injury and can vary by age, sex, and type of ABI (Figure 1). Most studies of TBI in juvenile rats have found greater BDNF protein concentration and *BDNF* mRNA expression up to 7 days after injury relative to sham or control animals [40,41,42]. In adult models, rats with TBI had higher *BDNF* expression acutely (within 6 h of injury) compared to shams, suggesting an upregulation of BDNF in the acute period, but similar or even lower *BDNF* expression compared to the sham group chronically after injury [43,44,45,46,47]. *BDNF* expression in adult models of ABI is associated with recovery in many of these studies [46,48,49]. Similarly, preclinical studies in adult models have shown therapeutic effects of BDNF treatment. Administering BDNF mimetics (7,8-dihydroxyflavone, R13) that better permeate the blood–brain barrier than native BDNF, or tropomyosin-related kinase B (TrkB) agonists that mimic the effects of BDNF at the TrkB receptor improve neurogenesis, metabolism, synaptic plasticity, and neurobehavioral recovery after TBI in adult rats [50,51,52,53,54]. In pediatric models of ABI, early work similarly demonstrates a likely association of BDNF with recovery. Juvenile rats with TBI had lower *BDNF* expression in the injured hippocampus at 14 days post injury relative to sham, corresponding with poorer cognitive functioning [55,56]. Most of these preclinical experiments limited their population to male rodents only. However, one study found region-dependent differences in BDNF expression after TBI with higher BDNF expression in the ipsilateral frontal cortex for males and higher BDNF in the contralateral hippocampus for females compared to sham [45]. Preclinical work evaluating the therapeutic effects of BDNF in models of TBI in immature animals of both sexes across the age spectrum is needed. 

In ischemic brain injury, BDNF responses depend largely on both the nature of injury (global cerebral ischemia following cardiac arrest vs. focal ischemia in pediatric stroke) and age [57]. In adult rats, global ischemia produced by either bilateral carotid artery occlusion or cardiac arrest (i.e., with total body ischemia) resulted in higher *BDNF* expression in the hippocampus compared to sham or controls in the 24 h after injury [58,59,60,61,62]. In pediatric models, however, a seminal study in juvenile (post-natal day 20–25) mice found lower *BDNF* expression 7 days after global ischemia due to cardiac arrest compared to the sham operation group. Critically, memory recovery and long-term potentiation in the hippocampus in that study were associated with an increase in *BDNF* expression from 7 days to 30 days in rats after cardiac arrest, not with neurogenesis, suggesting a potentially important role for BDNF in recovery [63]. In gerbils with global cerebral ischemia following transient bilateral carotid artery occlusion, *BDNF* expression was higher in injured animals relative to sham at 4 days after the insult in juveniles but not adults [64]. Notably, ischemic injury results in greater brain damage in gerbils than in rats, due in part to the lack of a circle of Willis in gerbils, which may explain the differences between animal models [65]. While there are few studies of pediatric ischemic injury after cardiac arrest, these initial studies suggest neuroprotective responses of BDNF in the post-ischemia stages of recovery and potentially key age-related differences for BDNF in the brain post injury.

While cardiac arrest in children results in global cerebral ischemia, pediatric stroke results in either focal or multi-focal ischemic injury. In stroke models, BDNF in the brain generally increases after injury. Most studies in adult stroke models found a greater BDNF concentration, *BDNF* mRNA, and uptake of BDNF by astrocytes acutely from 2 h to 7 days post injury compared to sham-operated rats [59,66,67,68]. In juvenile rats, greater BDNF-positive cells are found in the injured brain at both 7 and 14 days post-ischemic injury compared to rats with the sham operation [69]. However, future studies evaluating focal cerebral ischemia in juvenile rats at more acute time points are needed. Like TBI, evidence suggests therapeutic effects of BDNF in adult models of cerebral ischemia. Inhibiting BDNF blocks AMPA receptors and AMPA-mediated motor recovery following stroke in a mouse model compared to controls [70]. BDNF administration can have neuroprotective effects [71,72,73,74,75] and is associated with improvements in functional and behavioral outcomes [76,77,78], suggesting the importance of BDNF in recovery from ischemic brain injury in addition to TBI.

Finally, there is also preclinical evidence that increases in brain tissue expression of BDNF in ABI result from the differential modulation of *BDNF* after the insult [61], likely due to early changes in the methylome in response to injury. Preclinical studies in TBI models suggest the re-localization of DNA methyltransferase 1 (an enzyme that adds or removes methyl groups at cytosine residues) within reactive astrocytes and microglia as a likely mechanism [79,80]. Initial preclinical [79,81] studies show differential DNAm both acutely and months after TBI in adult models, but studies in pediatric preclinical models are lacking. Similarly, the dynamic temporal responses of BDNF in the injured brain warrant further study. Though most studies find that the spike in *BDNF* expression in the brain attenuates quickly following ABI, with some studies showing a return to control concentrations by 24 h [58,82] to one week post injury [83], changes in *BDNF* expression can continue for up to 20 weeks post ischemia [83].

**Table 1:** BDNF in preclinical models of ABI.

Reference	Type of Injury (TBI, Global Cerebral Ischemia, Stroke)	Brain Region from Which Sample Was Taken	Model	BDNF Concentration, Expression, Mimetics, or Genotype	Time Post Injury,	Results
Dyck et al., 2018 [40]	TBI	Motor cortex, prefrontal cortex	Juvenile rats post-natal day 27	Concentration	4 days	Rats with TBI had higher BDNF concentration in right and left motor cortex vs. sham
Griesbach et al., 2002 [41]	TBI	Hippocampus and occipital cortex	Juvenile rats post-natal day 19	Concentration, mRNA expression	24 h, 7 days, and 14 days	Rats with TBI had higher BDNF expression vs. sham at 24 h and 7 days in contralateral hippocampus and occipital cortex; higher BDNF concentration in occipital cortex and ipsilateral hippocampus at 7 and 14 days post TBI vs. sham
Rostami et al., 2014 [43]	TBI	Frontal cortex, hippocampus	Adult rats	Concentration, mRNA expression	24 h, 3 day, 2 weeks, 8 weeks	Lower BDNF expression in ipsilateral hippocampus and higher BDNF expression in contralateral hippocampus at 1 day, 3 days, and 2 weeks after TBI vs. sham; higher BDNF concentration in frontal cortex on days 1, 3, and 14 post TBI vs. sham
Hicks et al., 1997 [44]	TBI	Hippocampus	Adult rats	mRNA expression	1, 3, 6, 24, and 72 h	Higher BDNF bilaterally in dentate gyrus for 1 to 72 h post TBI vs. sham and in CA3 at 1, 3, and 6 h post TBI vs. sham
Chen et al., 2005 [45]	TBI	Hippocampus, frontal cortex	Adult rats	Concentration	4 weeks	Higher BDNF expression in ipsilateral frontal cortex for males vs. sham; higher BDNF in contralateral hippocampus vs. sham
Griesbach et al., 2009 [46]	TBI	Hippocampus, parietal cortex	Adult rats	Concentration	21 days	Rats with TBI had lower BDNF in ipsilateral hippocampus and injured parietal cortex vs. sham, but higher BDNF in contralateral parietal cortex vs. sham
Madathil et al., 2017 [47]	TBI	Hippocampus, cortex	Adult rats	Concentration	1 h, 6 h, 1 day, 2 days, 3 days, 1 week, 2 weeks	BDNF was higher in rats with TBI in the hours after injury vs. sham
Corne et al., 2019 [48]	TBI	Parietal lobe, hippocampus, amygdala, medial prefrontal cortex	Adult mice	mRNA expression	3 weeks	BDNF was lower in animals with TBI at exon IV vs. sham in injured parietal lobe
Thapak et al., 2023 [50]	TBI	Hippocampus	Adult rats	Mimetics, protein concentration (both mature BDNF and pro-BDNF, a precursor to mature BDNF)	8 days	Animals with TBI had lower mature BDNF vs. sham; rats treated with BDNF mimetic (R13) had greater mature BDNF and better cognitive function after TBI vs. controls
Agrawal et al., 2015 [51]	TBI	Hippocampus	Adult rats	Mimetics, concentration	6 days	Rats treated with BDNF mimetic (7,8-dihydroxyflavone) had less cognitive behavioral deficit and fewer cellular changes after TBI vs. controls; treatment group had similar cortical BDNF levels vs. controls
Wu et al., 2014 [52]	TBI	Parietal cortex	Adult mice	Mimetics, mRNA expression, and protein concentrations	1 day, 4 days	Mice treated with BDNF mimetic (7,8-dihydroxyflavone) had higher BDNF concentrations, improved survival, and reduced cell death after TBI vs. controls
Zhao et al., 2016 [53]	TBI	Hippocampus	Adult mice	Mimetics	2 weeks	Mice that received 7,8-dihydroxyflavone for 2 weeks after TBI had improved neurogenesis and dendrite arborization in the ipsilateral hippocampus vs. controls
Smith et al., 2023 [54]	TBI	Whole brain MRI scans	Adult rats	Mimetics	Up to 7 days post injury	Rats that received R13 had greater functional connectivity, and cellular and behavioral outcomes after TBI vs. controls
Schober et al., 2012 [55]	TBI	Hippocampus (ipsilateral)	Rat pups post-natal day 17	Concentration and mRNA expression	1, 2, 3, 7, and 14 days	Rats with TBI had lower BDNF protein vs. sham at 14 days
D’Cruz et al., 2002 [58]	Global cerebral ischemia	Hippocampus	Adult rats	Concentration	12 and 24 h	Rats with ischemia had higher BDNF concentrations vs. sham
Tsukahara et al., 1998 [59]	Global cerebral ischemia	Hippocampus, cortex	Adult mice	mRNA expression	2, 4, 8, 16, or 24 h	Mice with ischemia had higher BDNF mRNA in the hippocampus and cerebral cortex vs. controls
Dietz et al., 2018 [63]	Global cerebral ischemia	Hippocampus	Juvenile mice post-natal day 20–25	Concentration	7 days, 30 days	Lower hippocampal BDNF concentration vs. sham at 7 days in mice with cardiac arrest; no difference in BDNF in TBI vs. sham at 30 days
Yan et al., 2012 [64]	Global cerebral ischemia	Hippocampus	Juvenile gerbils and adult gerbils	Concentration	4, 7 days	BDNF expression was higher in injured animals vs. sham at 4 days after ischemia in juveniles but not adults
Li et al., 2020 [61]	Global cerebral ischemia	Hippocampus	Adult rats	Concentration transcript expression	48 h	Higher BDNF concentration vs. sham in CA3 and dentate gyrus; lower BDNF concentration vs. sham in CA1; higher BDNF mRNA vs. sham in CA1, CA3, and dentate gyrus at BDNF transcripts I, II, VI, and XI
Miyake et al., 2002 [66]	Stroke	Hippocampus	Adult rats	Concentration, mRNA expression	1, 3, 7 days	BDNF concentrations were higher in rats with ischemia vs. sham
Grade et al., 2013 [67]	Stroke	Striatum	Adult mice	mRNA expression	1, 2 weeks	Higher BDNF in ischemic striatum 1 week post injury vs. naïve mice
Lindvall et al., 1992 [62]	Global cerebral ischemia	Hippocampus	Adult rats	mRNA expression	10 min, 30 min, 2 h, 4 h, 24 h	Higher BDNF in dentate gyrus from 2 to 24 h in rats with ischemia vs. sham
Kokaia et al., 1996 [60]	Global cerebral ischemia	Hippocampus and parietal cortex	Adult rats	Concentration, mRNA expression	1, 2, 4, and 18 h (mRNA), and 6, 12, 24 h, or 1 weeks (protein)	Higher BDNF concentration at 6 h vs. sham in dentate gyrus and at 1 week in CA3; higher BDNF mRNA expression at 2 h in CA3 vs. sham
Madinier et al., 2013 [68]	Stroke	Cortex and hippocampus	Adult rats	Concentration, mRNA expression	4 h, 24 h, 8 days, 30 days	Higher mature BDNF in cortex vs. control; higher mature BDNF in hippocampus at 30 days vs. control
Cheng et al., 2020 [69]	Stroke	Ischemic penumbra	Juvenile rats (matured for 6–7 weeks)	Concentration, mRNA expression	7, 14 days	Higher BDNF concentration at 7 days vs. sham; higher BDNF expression in ischemic penumbra at 7 and 14 days post injury vs. sham
Clarkson et al., 2011 [70]	Stroke	Periinfarct cortex	Adult mice	Inhibition of BDNF	7 days	Mice with BDNF blocked have less AMPA-mediated motor recovery vs. controls
Zhang and Pardridge 2001 [71]	Stroke	Cortex	Adult rats	Mimetics	24 h	Rats treated with BDNF conjugate with a monoclonal antibody have smaller infarct size vs. controls
Zhang and Pardridge 2001 [72]	Stroke	Cortex	Adult rats	Mimetics	24 h, 7 days	Rats treated with BDNF conjugate have lower stroke volume at 24 h or 7 days after ischemia vs. controls
Shabitz et al., 2000 [73]	Stroke	Cortex	Adult rats	Mimetics	24 h	Rats with BDNF treatment have less neurological deficit and less stroke volume vs. controls
Yamashita et al., 1997 [74]	Stroke	Cortex	Adult rats	Mimetics	24 h	Rats with BDNF have lower infarct volume vs. controls; no differences in physiological measures vs. controls
Wang et al., 2023 [75]	Stroke	Cortex, cervical spinal cord	Adult rats	Mimetics, mRNA	28 days	BDNF-treated rats have better behavioral outcomes and greater corticospinal connections vs. controls
Zhang and Pardridge 2006 [76]	Stroke	Cortex	Adult rats	Mimetics	24 h	Animals treated with BDNF conjugate had greater motor outcomes and lower infarct volume vs. rats treated with BDNF alone
Ramos-Cejudo et al., 2006 [77]	Stroke	Cortex, serum	Adult rats	Mimetics, concentration	4 h, 7 days, 28 days	Recombinant BDNF-treated rats had better functional recovery and white matter repair markers at 28 days vs. controls
Alam et al., 2020 [78]	Stroke	Cortex	Young rats (3 months)	Concentration	6 weeks	Rats treated with p38 mitogen-activated protein kinase had higher BDNF after ischemia and greater functional recovery vs. controls
Kokaia et al., 1995 [82]	Stroke	Frontal and cingulate cortex, hippocampus	Adult rats	Concentration, mRNA expression	30 min, 2 h, 4 h, 24 h	Higher BDNF expression from 30 min to 4 h post ischemia vs. controls; no difference vs. controls at 24 h
Uchida et al., 2010 [83]	Stroke	Substantia nigra	Adult rats	mRNA expression	1, 2, 6, 20 weeks	Higher BDNF at 1 week and 20 weeks after ischemia vs. sham in neurons; greater BDNF released by astrocytes at 20 weeks vs. sham

## 3. BDNF in Clinical Studies of Pediatric ABI

### 3.1. BDNF Concentrations

Similar to both preclinical and clinical studies in adults [84,85], clinical studies in pediatric patients (see Table 2) show an initial increase in BDNF concentrations in plasma and cerebrospinal fluid (CSF) ~2 h after TBI, followed by a decrease in CSF BDNF concentrations at 24 h [86,87,88]. Two of the pediatric TBI studies failed to show a statistically significant association between BDNF concentration and dichotomized functional outcome. However, neither study was powered to appropriately test the hypothesis of an association (*n* = 14 children with TBI, *n* = 27 children with TBI in the two reports, respectively) [87,88]. In more recent studies of children with TBI and children with neurocritical-care-related conditions, higher BDNF plasma concentrations on day 1 (*n* = 177 children) [89] and BDNF serum concentrations at day 3 (*n* = 44) [90] were associated with better functional recovery, respectively.

Consistent with the pediatric TBI and neurocritical care studies above, two studies involving pediatric stroke in children with sickle cell disease also found higher BDNF plasma concentrations acutely after brain injury compared to other children with sickle cell disease or healthy controls (*n* = 8 with stroke, *n* = 40 with stroke, respectively) [91]. Importantly, preclinical studies in rats report conflicting evidence regarding whether BDNF concentrations in the periphery mimic cortical concentrations after stroke, when the blood–brain barrier remains largely intact with ischemia [92,93]. Thus, peripheral BDNF concentrations may not be correlated with CSF concentrations of BDNF, and assessments of both serum and CSF BDNF concentrations should be evaluated specifically in children after stroke. In pediatric cardiac arrest, an initial exploratory study of 42 children found no statistically significant associations of serum BDNF concentration at between 12 and 96 h post insult with dichotomized outcomes at six months [94]; however, once again, more extensive investigations with larger sample sizes are needed.

**Table 2:** BDNF in clinical studies of pediatric ABI.

Reference	Type of Injury (TBI, Cardiac Arrest, Stroke, Brain Mass, CNS Infection, or Inflammation)	Groups	Time Post Injury	BDNF Concentration, Genotype, or DNAm	Tissue (CSF, Serum, Plasma, Saliva)	Results
Tylicka et al., 2020 [86]	TBI	Children with mild concussion (−)LOC (*n* = 12); children with severe concussion (+)LOC (*n* = 17); and healthy controls (*n* = 13)	2–6 h	Concentration	Plasma	Higher plasma BDNF in children with mild head trauma (−)LOC and children with severe concussion (+)LOC 2–6 h post injury vs. healthy controls; BDNF concentration did not differ between children with mild vs. children with severe concussions
Chiaretti et al., 2003 [87]	TBI	Children with severe head injury (*n* = 14) vs. children with obstructive hydrocephalus (*n* = 12)	2 and 24 h	Concentration	CSF and plasma	Decrease in CSF BDNF concentration from 2 h to 24 h post injury
Chiaretti et al., 2009 [88]	TBI	Children with severe head injury (*n* = 32) vs. healthy controls (*n* = 32)	2 and 48 h after admission	Concentration	CSF	Higher CSF BDNF concentration in children with severe head injury vs. healthy controls; BDNF concentrations decreased in children with severe head injury from 2 to 48 h after admission
Pinelis et al., 2015 [89]	TBI	Children with TBI (*n* = 177)	1–3 days, 7–8 days, 14–15 days, 20–23 days, and 11–12 months	Concentration	Plasma	Decrease in BDNF concentration between days 1 and 3 post injury among mild and severe TBI; lowest BDNF concentration found at 1 day post injury in children with severe TBI and fatal outcomes
Madurski et al., 2021 [90]	TBI, cardiac arrest, stroke, brain mass, or CNS infection, or inflammation	Children with acquired brain injury (*n* = 44)	Admission days 0, 1, 3, 5, and day closest to hospital discharge	Concentration	Serum	Lower serum BDNF in children on day 3 of admission, day 5 of admission, and day closest to hospital discharge associated with greater functional impairment
Mahmoud et al., 2023 [91]	Stroke	Children with Sickle cell disease (*n* = 40) vs. healthy controls (*n* = 40)	During hospital admission	Concentration	Serum	Higher serum BDNF in children with SCD vs. healthy controls; higher serum BDNF in children with sickle cell disease associated with elevated transcranial doppler velocities
Kernan et al., 2021 [94]	Cardiac Arrest	Pediatric cardiac arrest patients (*n* = 42)	Twice within a 24 h period between 0 and 96 h and once at 196 h	Concentration	Serum	BDNF serum levels not found to be significantly associated with 6-month neurologic outcome
Treble-Barna et al., 2022 [95]	TBI	Children with TBI (*n* = 69) vs. OI (*n* = 72)	18 months	Genotype	Saliva	Allele status x injury group interactions associated with behavioral adjustment outcomes; within-group non-significant trends of poorer behavioral adjustment in Met carriers
Treble-Barna et al., 2022 [96]	TBI	Children with TBI (*n* = 69) vs. OI (*n* = 72)	18 months	Genotype	Saliva	Poorer verbal fluency functioning in Met carriers vs. Val/Val in the TBI group
Gagner et al., 2021 [97]	TBI	Children with mild TBI (*n* = 47), OI (*n* = 42), and typically developing children (TDC) (*n* = 56)	Any time point within 18 months	Genotype	Saliva	Val/Val mTBI associated with more internalizing problems vs. Met mTBI at 6 months post injury; Val/Val and Met mTBI groups associated with more internalizing problems vs. OI and TDC at 18 months post injury
Tuerk et al., 2020 [98]	TBI	Children with mild TBI (*n* = 52), OI (*n* = 43), and typically developing children (TDC) (*n* = 64)	Any time point within 18 months	Genotype	Saliva	Higher quality of life at 6 months post injury in Met carriers vs. Val/Val among TBI

### 3.2. BDNF Genotype

Only a handful of studies examining *BDNF* genotype have been conducted in pediatric ABI, with mixed results. In a concurrent cohort study of children with moderate-to-severe TBI or orthopedic injury but no TBI (OI; comparison group), the Val66Met met allele was associated with poorer longitudinal behavioral adjustment [95] and poorer long-term neuropsychological functioning [96] in children with TBI (*n* = 69) but not OI (*n* = 72). These results suggest that the Met allele—associated with reduced activity-dependent secretion of BDNF—may confer a risk for poorer neurobehavioral recovery from pediatric TBI. In contrast, the Met allele was associated with fewer internalizing problems (*n* = 145) [97] and better quality of life (*n* = 159) [98] in a cohort of children studied at 6 months after mild TBI, suggesting a protective effect. Reasons for these mixed results are unclear and require further investigation in larger cohorts and other etiologies of pediatric ABI.

Given the limited pediatric ABI studies, the adult ABI literature examining *BDNF* genotype merits discussion. There is a relatively extensive literature of candidate gene studies suggesting that the *BDNF* Met allele is associated with recovery from ABI in adults, especially following ischemic stroke [99]. Genome-wide association studies (GWASs), however, have not confirmed *BDNF*’s association with stroke recovery [100]. Similarly, the single GWAS of TBI recovery to date did not identify any genetic variants reaching genome-wide significance and *BDNF* was not included among the 13 genes with variants that reached a lower pre-specified sub-genomic statistical threshold [101]. The strengths and limitations of candidate genes vs. GWAS approaches in ABI are discussed below.

### 3.3. BDNF DNAm

An important consideration, and frequent criticism, for clinical studies of DNAm in ABI is the relevance of DNAm measured in peripheral tissues (most frequently blood) to brain function given that DNAm patterns are tissue-specific and reflect the local environment of each cell type. However, for clinical studies of BDNF in ABI, in particular, the use of peripheral leukocytes for DNAm measurement is justified. Although DNAm in brain tissue may provide more direct insights into the biology of brain function and pathology, acquiring in vivo brain tissue samples is not feasible outside of extraordinary circumstances—such as with resection of a cerebral contusion to treat severe intracranial hypertension. Further, to be useful as a clinical biomarker, it is essential that markers be identified in readily accessible tissues or cells, such as blood samples. A comparative analysis of genomic signatures of TBI revealed homology between the rodent hippocampus and peripheral leukocytes at gene, methylome, pathway, and network concentrations related to vascularity, cell integrity, and immune response [102]. Notably, *BDNF* was among the highest pathways with a shared homology between tissues. Further, rodent gene signatures showed a significant overlap with human genes of brain disorders identified by GWASs. These homologous changes across hippocampus and peripheral leukocytes likely reflect disruption of the blood–brain barrier in TBI, associated systemic inflammation, and potential changes in cell type composition in the systemic circulation mediated via the glymphatic system [102,103]. That study strongly supports the development of DNAm biomarkers of TBI using easily accessible peripheral blood leukocytes, as well as a focus on *BDNF*. Finally, taking an approach that collects DNAm data across the genome has the added advantage of being able to adjust for cell type heterogeneity, reducing potential confounding related to cell type–phenotype associations, which can help provide a greater biological understanding of ABI [39].

To date, all candidate gene studies of *BDNF* DNAm in ABI have been conducted in adults. *BDNF* DNAm in blood was associated with adult stroke outcomes, including global outcome, physical disability, cognitive dysfunction, anxiety, and depression, in several candidate gene studies [39,104,105,106,107]. Most studies reported hypermethylation in *BDNF* promoter regions in blood, suggesting lower *BDNF* expression, associated with poorer outcomes. In our study targeting *BDNF* DNAm in CSF over the first five days after severe TBI in 112 adults, trajectory analysis revealed low- and high-DNAm groups at two *BDNF* sites with suggestive associations with long-term neurobehavioral outcomes [108]. In contrast to the adult stroke studies, membership in the high-DNAm group was associated with better outcomes after severe TBI. The opposing direction of associations of DNAm with outcomes in this study as compared to the stroke studies may be explained by differences in the timing of sample collections (weeks to months post stroke vs. the first five days post TBI) as well as evidence for a negative correlation between CSF and peripheral *BDNF* levels [84]. Beyond candidate studies, three epigenome-wide association studies (EWASs) have been conducted in adult TBI [109,110,111]; however, *BDNF* DNAm did not reach epigenome-wide significance in any study. While there are no published candidate *BDNF* DNAm studies in pediatric ABI, two published studies using an EWAS approach have examined DNAm in association with recovery from mild TBI [112,113]. In a blood-based EWAS of 17 children with mTBI vs. 18 healthy controls, one CpG site in the *BDNF* gene was among the 449 differentially methylated (hypomethylated) sites reaching epigenome-wide significance [112]. In a larger saliva-based EWAS of 110 children with mTBI and 87 healthy controls, *BDNF* DNAm was not significantly associated with quality of life or persistent post-concussive symptoms [113]. A candidate study of *BDNF* DNAm in association with neurobehavioral recovery following moderate-to-severe pediatric TBI is ongoing [114].

## 4. The Potential of BDNF as an ABI Biomarker Responsive to Environmental Influences

**Figure 2:**
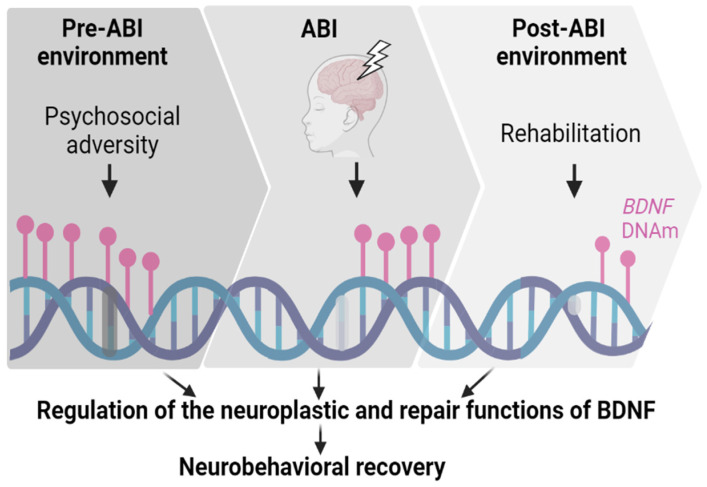
The potential role of brain-derived neurotrophic factor (BDNF) DNA methylation (DNAm) in neurobehavioral recovery from acquired brain injury (ABI). Pre-injury environmental factors (e.g., psychosocial adversity) may downregulate BDNF by increasing BDNF DNAm. ABI itself alters BDNF DNAm, leading to an increase in BDNF after injury. Post-injury factors (e.g., rehabilitation) can also decrease BDNF DNAm, leading to an increase in BDNF expression. This pre- and post-ABI epigenetic regulation of BDNF may influence neuroplasticity and ultimately neurobehavioral recovery after injury.

The unique potential of BDNF as a biomarker of recovery from pediatric ABI is underscored by its responsiveness to environmental influences, especially as mediated by changes in DNAm. It is well established that environmental factors influence recovery from ABI, though their underlying mechanisms are poorly understood. One of the most significant examples is the well-documented outcome disparities (i.e., poorer neurobehavioral recovery) among children facing greater psychosocial adversity, including low socioeconomic status and greater family dysfunction, even after adjusting for pre-injury functioning [7,115,116,117,118,119,120,121,122]. A second major environmental factor with potential to alter recovery trajectories is the quality and quantity of rehabilitative therapies, though research into these effects remains in its infancy [123,124,125]. As we review below, given the role of *BDNF* DNAm in the biological embedding of the psychosocial environment, and its responsiveness to interventions, we posit that *BDNF* DNAm may confer risk or protective effects on recovery after pediatric ABI by regulating the neuroplastic and repair functions of BDNF (see Figure 2).

### 4.1. Psychosocial Environment

Preclinical and clinical studies in non-brain-injured animal models and individuals suggest that *BDNF* DNAm is involved in the biological embedding of the psychosocial environment with downstream effects on brain function. Preclinical models of early-life caregiver maltreatment have shown alterations in BDNF DNAm in the medial prefrontal cortex, amygdala, and hippocampus [126]. These changes in methylation have been identified within 24 h of caregiver manipulation, can persist through adolescence and into adulthood, and have been associated with cognitive dysfunction [126,127,128]. Psychosocial environmental factors examined in association with *BDNF* DNAm in human studies have included a multitude of trauma/stress exposures [129,130,131,132,133], as well as neighborhood-level socioeconomic disadvantages [133]. Of these, both candidate studies and EWASs report differential *BDNF* DNAm [39], as well as associated effects on downstream neurobehavioral outcomes such as depression and anxiety [134,135]. While several studies have begun to examine DNAm in clinical studies of ABI (reviewed above), no published studies to date have examined *BDNF* DNAm in association with both the psychosocial environment and recovery.

### 4.2. Rehabilitative Therapies

Emerging evidence suggests that BDNF is responsive to rehabilitation interventions after ABI. These studies are largely in animal models and most commonly evaluate physical activity as an intervention via voluntary wheel running or treadmill training (forced exercise that is more tightly controlled under experimental conditions). Early studies showed that voluntary wheel running following TBI upregulated *BDNF* expression and had corresponding improvements in cognition, but suggested a therapeutic window of rest following injury [136,137,138,139,140]. More recent studies, however, have shown favorable improvements in behavioral and neurobiological responses to injury (including BDNF response) with exercise starting early after TBI, but have also found that the intensity and duration of exercise matter early in recovery [141,142,143,144,145,146,147,148]. Several studies have shown that BDNF mediates this association between physical activity and TBI recovery [137,138,149]. In animal models, physical activity decreases *BDNF* DNAm, upregulates hippocampal BDNF, and initiates synaptic plasticity pathways to ultimately improve neurobehavior [138,150]. In preclinical models of stroke, aerobic exercise improves neurobehavioral symptoms, such as depression, by regulating *BDNF* expression in both adult and juvenile animals [69,151,152,153,154,155,156,157,158]. A recent study evaluating aerobic exercise in rats with global cerebral ischemia found that exercise promoted neuron repair and survival, mediated in part by BDNF [159]. Furthermore, some studies have shown that pre-conditioning, or exercising prior to brain injury, can improve BDNF concentrations post injury and improve recovery after ABI, as well [160,161]. In initial clinical studies of adults post stroke, individuals who performed physical exercises had increased BDNF concentrations compared to pre-intervention [162,163] and, in one study, BDNF serum concentrations were associated with cognitive recovery [163]. In other non-brain-injured adult populations, cognitive improvements found with physical activity are mediated in part by epigenetic changes in *BDNF* expression, demonstrating the impact of BDNF on rehabilitation and recovery [20,104,164,165].

Similar rehabilitation techniques, such as task-specific training [166] and enriched environments that mimic clinical rehabilitation environments, have also been shown to improve recovery from experimental ABI in animal models [167,168,169]. Early environmental enrichment counteracted the detrimental effects of prenatal alcohol exposure on behavior, potentially mediated by the fourfold increase in BDNF expression seen with environmental enrichment [167]. Several papers in preclinical stroke studies have found that BDNF plays an integral role in the relationship between rehabilitation and recovery [170]. After stroke, mice in an enriched environment have higher BDNF concentrations and improved cognitive performance compared to mice housed in a standard environment [171]. Importantly, blocking BDNF for 28 days after stroke in rats negated the effects of rehabilitation on functional recovery, suggesting the key role of BDNF in stroke recovery [172]. These findings suggest that *BDNF* expression could inform therapeutic decision making through the monitoring of therapeutic effects for children with ABI [166].

## 5. *BDNF* DNAm as a Modifiable Therapeutic Target

Finally, the therapeutic potential of *BDNF* DNAm is further highlighted by the fact that DNAm is modifiable. The pharmacologic inhibition of DNAm provided neuroprotective effects against ABI in adult animal models [173,174,175]. Similarly, non-pharmacologic [165,166,167,168,169,176] therapies, such as environmental enrichment and physical activity designed to mimic rehabilitation settings, ameliorated changes in DNAm and BDNF expression after neonatal ABI and in adults with post-traumatic stress disorder. Drugs targeting the methylome are already used in different diseases, such as cancer [177,178,179], suggesting the therapeutic potential of DNAm modification. Though small peptide BDNF mimetics, such as 7,8-dihydroxyflavone and R13, have shown therapeutic potential in animal models, the studies of pharmacokinetics and bioavailability are still in initial stages of preclinical testing [180]. Given that there are currently no neuroprotective therapies proven to improve recovery from pediatric ABI, the identification of potential therapeutic targets, such as the methylome, in this population is critical.

## 6. Discussion

We summarize existing evidence from both preclinical and clinical studies suggesting that BDNF holds significant potential as a biomarker of survival and recovery from pediatric ABI. Preclinical and clinical studies converge to show alterations in BDNF concentrations, gene expression, and DNAm after ABI and associations of these changes with neurobehavioral recovery. Moreover, we review emerging evidence in ABI and non-brain-injured populations suggesting that BDNF is involved in the biological embedding of the psychosocial environment, is responsive to rehabilitative therapies, and is potentially modifiable. These unique features set BDNF apart from traditional biomarkers, suggesting that BDNF may be reflective of and responsive to both pre- and post-injury environmental influences. The discovery of such a biomarker holds the potential to revolutionize pediatric ABI management by identifying novel targets for therapy development, improving prognostic tools, and aiding therapeutic decision-making.

There remain many gaps and opportunities for additional research. While it appears that there are alterations in BDNF after ABI, the timing and direction of the BDNF response vary across studies, possibly related to age, type of brain injury, time since injury, tissue, genetics, or other confounders. Additional studies are needed to clearly understand these dynamics. As is the case in many conditions, knowledge of the role of BDNF in ABI in the pediatric literature lags behind what has been reported after ABI in adults. It will be important to study the potential of BDNF as a biomarker in pediatric ABI, specifically as adult findings cannot be generalized to children given differences in etiologies, mechanisms, and biological responses to brain injury, ongoing and rapid brain development, and the unique psychosocial environments of children. Additionally, many of the existing studies had relatively small sample sizes often due to the lower prevalence of ABI in single-center studies of children versus adults. Multicenter studies will be essential to obtain sufficiently large sample sizes for more rigorous and appropriately powered analyses. Finally, while several candidate gene studies have identified significant associations of the *BDNF* genotype and DNAm with ABI recovery, several GWASs/EWASs have not confirmed this association. GWASs/EWASs are rigorous approaches that may be used to discover genes with the largest main effects in association with a phenotype, but these approaches have their own limitations, especially in investigations of complex behavioral phenotypes influenced by environmental and developmental factors [181], like recovery from ABI. Candidates and EWAS/GWAS investigations should be completed concurrently with deep phenotyping and in vivo physiological and intermediate phenotype measurements to understand biological mechanisms [181]. In addition, while EWASs are essential for the discovery of DNAm sites with the largest effects, EWAS approaches are currently impractical for the clinical setting primarily due to cost and time constraints. To aid in the clinical translation of EWAS results, the most compelling DNAm signals from EWASs should be tested for consistency using targeted pyrosequencing, which measures DNAm from a select number of sites rather than the wider methylome, enhancing the potential for clinical point-of-care application. Collaborative, large-scale investigations will be essential for the translation of significant genetic and epigenetic findings into biomarkers for clinical use.

The potential of BDNF as an ABI biomarker responsive to the psychosocial environment has yet to be explored in clinical studies of pediatric ABI. As has been demonstrated in non-brain-injured animal and human studies, *BDNF* DNAm is involved in the biological embedding of the psychosocial environment. Investigation of the associations among pre- and post-ABI psychosocial environments, *BDNF* DNAm, and recovery could reveal a biological mechanism to partially account for the unexplained heterogeneity in ABI recovery and the neurobehavioral outcome disparities associated with psychosocial adversity. Targets for such studies could also include other genes with dual roles in the biological embedding of the psychosocial environment and response to TBI, including interleukin-6 (IL-6), interferon-gamma (IFN-γ), and tumor necrosis factor alpha (TNF-α), among others [182,183].

Similarly, most studies evaluating the response to rehabilitation (e.g., physical activity or environmental enrichment) are preclinical studies, with a few clinical studies in adult stroke populations [162,163]. Studies evaluating associations between physical activity or rehabilitation and BDNF in pediatric ABI are critical to provide a biomarker to track the response to interventions and aid in developing new interventions to improve ABI recovery. Other growth factors may also play a role in rehabilitation and recovery after brain injury, often interacting closely with BDNF. Insulin-like growth factor-1 (IGF-1), glial-derived growth factor (GDNF), and vascular endothelial growth factor (VEGF) have also been implicated in mediating the effects of rehabilitation after injury [64,184,185]. Similar to BDNF, IGF-1 also plays an important role after brain injury [186] and studies have shown that IGF-1 may mediate the exercise-induced regulation of BDNF and cognitive improvements after brain injury [185,187,188]. Identifying the key molecular pathways involved in brain injury and recovery and their roles in rehabilitation will be critical to guiding and optimizing current and future therapies after ABI.

There are limitations that can complicate data interpretation when comparing studies. A factor that can contribute to the variation in concentrations of BDNF in blood or CSF relates to differences in assays used between studies. BDNF concentrations in biological samples are commonly assessed using ELISA, multiplex assays, or Western blot. Differences in the epitope targeted by the detecting antibody across these assays can vary, as can the accessibility of the antibody to the epitope. For example, linearization of the BDNF protein as assayed by Western blot can enhance antibody binding compared to assessment of the native BDNF molecule in blood. Similarly, cross-reactivity with other analytes in multiplex assays can in some cases be observed and can impact concentrations. Differences in concentrations of other molecules, such as TNFα, between ELISA and multiplex assays have been well described [189]. Finally, if tissue samples are assessed for protein concentrations using immunohistochemistry, further variability can be produced, once again related to the accessibility of the antibody to the epitope and cross-reactivity. And similarly, if ELISA is used to assess homogenates of tissue samples, the method of tissue processing and solubilization can also impact levels [189].

There are many potential clinical and research implications of BDNF as a dynamic and potentially modifiable pediatric ABI biomarker. Accounting for the additional heterogeneity in ABI outcomes via a biomarker that is reflective and responsive to environmental influences could result in the provision of more accurate prognostic information for patients and families, as well as more powerful studies of intervention efficacy. Additionally, BDNF could serve as a therapeutic target for future interventions, both pharmacological and non-pharmacological (e.g., physical activity). Finally, the responsivity of BDNF to rehabilitation suggests the potential for aiding in therapeutic decision making by monitoring a child’s recovery and response to therapies.

## 7. Conclusions

BDNF holds exciting potential as a pediatric ABI biomarker that could revolutionize pediatric ABI management. It will be important for future work to build upon the foundational evidence reviewed here, integrating knowledge gained across preclinical and clinical studies (adult and pediatric) from different etiologies of ABI, and in relevant fields outside of ABI.
